# On the Different Forms of Insanity in Relation to Jurisprudence

**Published:** 1843-07

**Authors:** 


### ART. V.

1. *On the different forms of Insanity in relation to Jurisprudence.* By JAMES COWLES PRICHARD, M.D.—*London*, 1842. 8vo, pp. 243.
2. *The Plea of Insanity in Criminal Cases.* By FORBES WINSLOW, M.R.C.S.—*London*, 1843. 8vo, pp. 78.
3. *Criminal Jurisprudence considered in relation to Cerebral Organization.* By M. B. SAMPSON.—*London*, 1843. 8vo, pp. 147.
4. *Commentaries on some Doctrines of a dangerous tendency in Medicine.* (Comm. III.—*On some Important Questions relating to Insanity, both in a Medical and Legal point of view.*) By SIR ALEXANDER CRICHTON, M.D. F.R.S.—*London*, 1842.
5. *Report of the Trial of Daniel M'Naughten for the wilful Murder of Edward Drummond, Esq.* By R. M. BOUSFIELD and RICHARD MERRETT.—*London*, 1843. 8vo, pp. 78.
6. *M'Naughten. A Letter to the Lord Chancellor upon Insanity.* By J. Q. RUMBALL, Esq. M.R.C.S.—*London*, 1843. 8vo, pp. 35.
7. *On the Amendment of the Law of Lunacy; a Letter to Lord Brougham.* By a Phrenologist.—*London*, 1843. 8vo, pp. 39.

THE great interest which is taken by the profession and the public in the medical jurisprudence of insanity is we think sufficiently evinced by the number of publications which have lately appeared on the subject. Some heinous crimes have been of late years perpetrated, which have called especial attention to the subject of homicidal insanity, and the most recent case, namely that of M'Naughten, has in some respects modified the old legal doctrine of responsibility. Several of the works placed at the head of this article have been called forth by the medical evidence given on this occasion, as to the irresponsibility of the insane. Before noticing these we shall first proceed to examine a work of more general and permanent interest.

I. The small volume by Dr. Prichard may be regarded as an abridgment of the treatise on Insanity, by the same author, which we reviewed in our Thirteenth Number. It is written in a more popular style, and is evidently intended for the use of commissioners and juries, engaged in the investigation of questions relative to the sanity or insanity of individuals. This, indeed, the author professes to be his object in the preface. A very large portion of the treatise is occupied with a description of the characters peculiar to the various forms of insanity, and of the means of distinguishing one form from another. Every point relative to treatment and the medical management of the insane is of course avoided, as not being within the scope of the work. We pass over the author's analysis of the conflicting opinions of lawyers on mental unsoundness, this subject having been already fully treated in a previous number, (No. XIX, p. 120.) It will be our object to comment in this place on those parts of the treatise only, the subjects of which were but slightly noticed in our former article on the medical jurisprudence of insanity.

Dr. Prichard believes it to be the settled doctrine of the English courts at present, that there cannot be insanity without delusion, or, as it is otherwise expressed by physicians, without illusion or hallucination, that is, without some particular erroneous conviction impressed upon the understanding, the affected person being otherwise in possession of the full and undisturbed use of his mental faculties. This, he observes, is the doctrine of partial insanity, so that a man is supposed to be mad upon one point, and sane on every other particular, a state in itself most incredible. The only admissible view of partial insanity is that which was taken by the present Lord Chancellor (Lyndhurst.) "It is that the mind is unsound, not unsound on one point only, and sound in all other respects; but that this unsoundness manifests itself principally with reference to some particular object or person." (p. 16.) The author further remarks that mental derangement in almost every case not only involves a disordered exercise of the intellectual faculties, but extends even further than the understanding, and implicates more remarkably the moral affections, the temper, the feelings and propensities; that it affects in reality the moral character even more than the understanding.

The author devotes considerable space to the subject of moral insanity, by which we are to understand a disorder affecting only the feelings and affections, or what are termed the moral powers of the mind, in contradistinction to the powers of the understanding or intellect. In our notice of Dr. Prichard's former work, (No. XIII, p. 6,) we expressed the opinion that there is some difficulty in admitting the existence of any form of moral insanity, disjoined from some degree of mental infirmity, less perceptible in the slighter, but manifest in the more severe cases. Esquirol is also opposed to this view of Dr. Prichard's; he does not consider it possible that the intellect can be in a state of integrity in what is called moral insanity. The truth probably is, that the understanding is disturbed in all the cases, only more in some and less in others. Our opinion is not shaken by the cases cited by Dr. Prichard. We may find it in some instances difficult to detect the evidence of any "fixed notion" which influences a man's conduct on the existence of any "delusive ideas;" but still we believe that there is in every case of true insanity some latent disorder of the intellectual powers.

In a subsequent chapter, when treating of the irresponsibility of madmen, the author complains, "That the attention of those who have hitherto investigated cases of insanity has been too much directed to the particular error which clouds the understanding, or to the disordered state of the intellect, or judging and reasoning powers; whereas, in reality, it is of the moral state, the disposition and habits of the individual concerned, that the principal account ought to be taken." (p. 175.)

We admit that there is some truth in this observation, and that time is often wasted by a physician in endeavouring to detect some absurd delusion; but, nevertheless, this is often the only way by which we can arrive at a knowledge of the course on which the perversion of the moral feelings and affections depends. If all physicians admitted, like Dr. Prichard, that moral insanity was perfectly independent of and distinct from what is called by him intellectual insanity, then there might be some reason for censuring this limited method of inquiry into the state of the intellect; but as the common opinion is rather opposed to this view, we conceive that a man who looked only to the habits and disposition of a party would often be led into error.

We have already seen that the author is himself a strong opponent of the doctrine of partial insanity, as applied to the intellectual powers. He believes that in all such cases there is more or less disturbance of the whole of the intellectual faculties. But we doubt whether the facts adduced in support of this view be any stronger than those which tend to show that there is no form of moral insanity without some trace of perversion of the intellect. We are glad to perceive that the author, notwithstanding his belief in the nonexistence of partial insanity, as it is commonly understood, is opposed to the general practice of placing such persons under restraint on the most trivial pretexts.

> "A man who, like Count Swedenborg, labours under a harmless hallucination, or fancies that he converses with angels and meets Moses in Cheapside, ought not to be confined in a lunatic asylum. But it will generally be found on inquiry, that persons who have illusions are otherwise incapable of exercising civil rights." (p. 67.)

All we can say is, if such persons are shown to be incapable of performing their civil duties, let them be placed under proper restraint or interdiction; but it has happened in many cases, and in our view most unjustly, that the incompetency to manage affairs has been a hasty inference from the proof of the existence of some foolish notion in the person's mind. How far this notion affected his actions was not so much a matter of inquiry as the mere proof of its existence. We have known an instance where a lady was interdicted in consequence of her entertaining the belief, in which she persisted before the commission, that the Duke of Wellington was in love with her, and was about to marry her. We could not ascertain from the evidence that this delusion had operated injuriously upon her general conduct, or that it had thrown her affairs into disorder.

In some cases of this kind the object of an inquiry has been almost defeated by the extreme difficulty which existed in detecting any delusion in the mind of the alleged lunatic. When this delusion was ultimately laid bare by the ingenuity of a physician, after a cross-examination of some hours, the result has been looked upon as a matter of triumph and justification; but, as Dr. Conolly has remarked, in speaking of cases of this kind, one point seems to have been wholly lost sight of; namely, what injury could have resulted to the patient or his friends from the presence of a delusion in his mind, over which he had such control, that it required many hours and much ingenious questioning even to detect its existence? Of course we admit that these cases require close watching, since the existence of any kind of delusion indicates more or less of a disordered state of the mental faculties.

In treating of homicidal insanity, and of the means of distinguishing the insane homicide from the sane murderer, Dr. Prichard observes:

> "The act of homicidal insanity is different in its nature and moral causes from that of the murderer. Men never commit crimes without some motive; the inducement which leads them to an atrocious act is of a kind which other men can appreciate and understand, (?) though they do not sympathise with them. Jealousy, hatred, and revenge excite some; others are moved by the desire of plunder, of getting possession of money or property. The act of a madman is for the most part without motive, and even contrary to all the imaginable influence of motives. .......... There have been some instances in which the perpetrator of insane homicide has been excited by a real monomaniacal delusion, a firm and strong belief that he acted under a divine command." (p. 127.)

In our former article, the subject of homicidal insanity was examined chiefly as to one of its characters, namely the existence of *delusion*. We said little about *motive;* and it is therefore now our purpose to make some remarks on this assumed criterion of sanity in the commission of crime. The question is the more important, because since the article referred to was written this very criterion has been made a prominent subject of discussion in relation to the attempts made on the life of the sovereign, as well as in respect to certain other atrocious crimes committed on private individuals. From the above paragraph, Dr. Prichard evidently assumes that sane men never commit a crime without an apparent motive; and that an insane person never has a motive, or one of a delusive nature only, in the perpetration of a criminal act. If these positions were true, we apprehend that there would be no difficulty whatever in distinguishing a sane from an insane criminal; but in practice the rule is only applicable to a very limited extent, and is often liable to mislead.

The fallacy of trusting to such a criterion must, we think, be apparent by examining the question, whether the responsibility of a criminal is to depend *on the discovery or non-discovery of a motive* for the act of which he has been guilty. We put the question in this, the plainest, light, because, in point of law, the non-discovery would be taken as synonymous with the non-existence of motive; and the prisoner in such a case would receive the benefit of the doubt incurred by deciding on his responsibility according to this false criterion. "De non existentibus et non apparentibus eadem est ratio" is a well-known rule of law.

Georget and others who, with Dr. Prichard, have advocated this view, have forgotten that there is a very wide distinction between the non-existence and the non-discovery of a reasonable motive for the commission of a crime. It is undoubted that a motive may exist for the most atrocious criminal act without our being able to discover it. This is abundantly proved by the numerous recorded confessions of criminals before execution, in cases where, until these confessions had been made, no possible motive for the perpetration of the crimes had appeared even to the acutest minds. Two persons are seen together on friendly terms; one is afterwards found murdered, and circumstances inculpate the other. No motive appears for the crime, for he makes no confession, and no eye witnessed the transaction. Are we, therefore, to acquit the accused and pronounce him irresponsible? In the case of Courvoisier, who was convicted of the murder of Lord William Russell in June 1840, it was the reliance upon this fallacious criterion, before the secret proofs of guilt accidentally came out, that led many to believe he could not have committed the crime; and the absence of motive was urged by his counsel in defence as the strongest proof of the man's innocence. It was ingeniously contended: "that the most trifling action of human life had its spring from some *motive* or other." The sophistry of this position is very evident. Allowing that all the actions of men proceed from motives, it is not always in our power to discover them; and therefore it would be in the highest degree absurd to rest the innocence or responsibility of an accused person on so feeble a ground.

On the trial of Francis for shooting at the queen, one main ground of defence urged in proof of the prisoner's irresponsibility by his counsel, was that the prisoner had *no motive* for his act. In the language of Dr. Prichard, it was argued, that "*men never commit crimes without some motive*." It is thus we see the danger of giving circulation to unsound medico-legal axioms. We shall here quote the observations of the present solicitor-general on the occasion of this trial, in reference to this question of motive, since they strike at the root of the false principle involved in this assumption :

"This doctrine about motive is of a most dangerous character, and must be very guardedly received. It is very difficult for you (the jury), very difficult for any well-regulated minds, not accustomed to contemplate the workings of iniquity, to discover the motives for crime. What motive instigated the execrable assassin in Paris, who shot at his king and deluged the streets with blood by means of his infernal machine? Did any one ever hear propounded in a court of justice a doctrine that would lead to such dangerous consequences to society as that you must ascertain the motive before you convict of the crime?"

It is an undoubted fact, that crimes are sometimes committed without any apparent motive by sane individuals who were at the time perfectly aware of the criminality of their conduct. Some years ago there were, in this metropolis, several miscreants who went about stabbing females with knives or dirks in the dusk of the evening, inflicting thereby dangerous wounds in the groin and thighs. The females were perfect strangers to them ; there was no attempt at robbery, no demand for money, and in short no sort of motive could be discovered, on their apprehension, for this most wantonly malicious conduct. That they were fully aware of the crime was proved by their endeavouring to escape immediately after its perpetration. No evidence of insanity or delusion could be discovered about them. They had nothing to say in their defence. The crime was soon checked by very properly making them responsible, notwithstanding the absence of motive, and by inflicting on them severe punishment. No case can more strongly show the impropriety of resting the question of responsibility on general rules of this kind.

We are willing to admit, that an absence of motive may, in some instances, favour the view of irresponsibility for crime. These cases are, where there are other strong evidences of insanity ; but what we strongly protest against is that insanity and irresponsibility should be inferred to exist whenever a motive for a crime cannot be detected.

But on the other hand—Does the discovery of what may be called a rational motive for a crime like murder, such as revenge for real injuries received, necessarily indicate that the party is sane and that he should be held responsible? Dr. Prichard does not exactly touch upon this question, although his language evidently implies that the existence of such a motive should render the party responsible. It may, however, be fairly objected to the exclusive application of this doctrine, that attendants have been on several occasions murdered by lunatics, where the latter have experienced violence or ill-treatment at their hands. A case of this kind is related by Dr. Haslam, and will be given hereafter. In answering such a question we are bound to consider, whether in insanity all those passions and feelings are annihilated which so often supply motives for crime to some men. It is, we believe, an undisputed fact that the insane are susceptible of revenge; but they are never known to commit crime for the sake of robbery or for the concealment of another crime.

Thus then, admitting as a general principle, that real motives for crime are only imputable to the sane, we must contend that the absence of all apparent motive is neither to be taken as absolute evidence of insanity nor as a plea for irresponsibility. And again, the occasional discovery of what appears a rational motive for crime, such as revenge or hatred, must not lead us to suppose that the individual is necessarily sane and responsible for his act. We believe that great mischief is done both in and out of the profession, by laying down such criteria for the distinction of homicidal insanity. It would be far better to admit that there are no well-founded means of diagnosis. Each case must be judged of by itself: it cannot be determined by any legal or medical rules derived from a few cases, or founded on a narrow circle of observations. If the crime has proceeded from the usual incentives, if the criminal is aware of the real effect of a plea of insanity, and exerts himself to create a belief in others that he is really insane, there can be no pretence whatever for exculpating him, even although we may be ignorant of his real motive in committing it, or the act itself may be "contrary to all the imaginary influence of motives."

We agree with Dr. Prichard in considering " that *suicide* is not always the result of insanity. (p. 134.) We need not enter into his reasons for this opinion, as the subject has been already sufficiently discussed in previous articles.

In regard to *lucid intervals* the author thinks that we ought never to convict for a crime committed during this state, " because there is every probability that the individual was under the influence of that cerebral irritation which makes a man insane." (p. 201.) We consider that there is great truth in this remark, and we do not now find that juries are so ready to convict a prisoner who is admitted to have been insane within a short period of time before the perpetration of the criminal act with which he is charged.

In treating of imbecility, the author adopts the artificial degrees of this state proposed by Hoffbauer; although he substantially admits that the application of any such divisions to practice would be less successful than the method at present pursued by English lawyers. To this chapter there is attached but a very slight and imperfect notice of the celebrated case of Miss Bagster, of which we have elsewhere spoken. (No. XIX, p. 155.) We much doubt whether the author can have read the excellent report of this case, published in the Medical Gazette, (vol. x. p. 519;) for we find that he completely mistakes the evidence of Dr. Morison and Dr. Haslam in saying: " Drs. Morison and Haslam had both visited her, and were disposed to consider her imbecile or idiotic." (p. 227.) What Dr. Morison did say on this occasion is as follows: " Her (Miss Bagster's) incompetency to manage her affairs, arises not from unsoundness of mind, but from ignorance," and Dr. Haslam's evidence stands thus in the report: " She (Miss Bagster) is not a lunatic, she is not an idiot, nor is she of unsound mind." We feel disposed to think that our English author has taken his account from the American writer, Dr. Ray. It is to be regretted that this work should thus have been made the means of circulating an error with respect to one of the most important medico-legal cases which have occurred in modern times.

In concluding our notice of this volume, we must repeat a complaint which we made in reviewing the treatise by the same author a few years since. His references are almost invariably made to foreign documents. It is true that some of the best works on insanity are in the French and German languages; but we do not see why, if these were consulted as authorities, his illustrations should not have been derived from English cases. There is no want of cases in England to illustrate every department of the medical jurisprudence of insanity. Not a single year passes without many curious and intricate questions relative to the civil and criminal liabilities of lunatics coming before our courts of law. Dr. Prichard entirely neglects these, and takes up the old and well-known cases of Esquirol and others. The important case of Oxford, tried for shooting at the queen, is not even noticed; that of Francis for the same offence probably had not commenced until the greater part of the work had been written.

On the whole, however, we do not hesitate to say that this will be a useful manual to those whose avocations do not allow them sufficient time to go deeply into the subject of insanity. It may be considered as containing the essence of the author's former work, the reputation of which is sufficiently established to ensure a wide circulation to the present treatise.

II. The essay of Mr. Winslow is chiefly confined to the subject of the irresponsibility of the insane for criminal offences. While the author presents us with nothing new, he gives a very fair popular outline of the modern doctrine of homicidal insanity. The dicta of different judges, from Lord Hale downwards, are cited to show that the views of lawyers are wholly unsettled on this subject. The only conclusion to be derived from an examination of these authorities, is that, in cases of insanity, "the law looks to the capability of distinguishing between right and wrong; of the person knowing that the crime of which he stands accused is an offence against the laws of God and man." (p. 9.) In plain language, the conclusion amounts to this: if a man knows that what he is doing is contrary to law, he is responsible for the act, otherwise not: right and wrong must then here stand, as Lord Brougham has suggested, for legality and illegality. The difficulty lies, however, in applying such a test. How are we to discover what a man's views are of the legality or illegality of the act which he is perpetrating? We cannot take it from his confession; and if we take it from circumstances, we are very liable to be deceived. Bellingham did not admit that he had done wrong in shooting Mr. Perceval; and there was every reason to believe that he was insane: he was however convicted and executed. Martin, the incendiary, admitted that he knew he was doing wrong, according to the law of man, when he set fire to York Cathedral; he knew that the act was illegal, but he said he had the command of God to do it. There was no doubt that it was perpetrated under a delusion, and he was acquitted. Thus, then, it appears from this case that a man may have a full conviction that the act which he is perpetrating is illegal, and yet be held irresponsible. Some homicidal monomaniacs have committed murder, in order that they might suffer death according to law, considering that they were forbidden to destroy themselves. They must, in these cases, have been fully aware that the crime which they were about to perpetrate was contrary to law, and have actually looked forward to the punishment which they conceived would deservedly follow the offence. For a remarkable instance of this kind, see No. XIX. of this Journal, p. 144. The case of Hadfield, who was tried for shooting at George III., furnishes another striking example of the existence of insane delusion, coupled with a knowledge of the consequences of the act which he was about to commit. He knew that in firing at the king he was doing what was contrary to law, and that the punishment of death was attached to the crime of assassination ; but the motive for the crime was that he might be put to death by others; he would not take his own life. The legal test, then, here falls short of what is necessary for justice. A consciousness that the act committed is contrary to the law of God and man may exist, and yet the person be held irresponsible. Hence this mode of testing criminal responsibility, withou taking into consideration numerous other circumstances, is incorrect. Cases occur in which it is impossible to act upon it.

"Many able writers on jurisprudence," says Mr. Winslow, "have maintained that in no instance where insanity is established ought the person so unhappily affected to be considered a responsible agent. So little, it is said, is known of the condition of the mind, even in cases of decided monomania, or delusion upon one point only, that it is impossible to form anything like a correct notion of the condition of the other mental faculties, or to ascertain with anything like precision to what degree they may be occasionally implicated in the disorder, and thus drive the person to the commission of capital offences." (p. 12.)

While he thus fairly represents his views of this difficult question, Mr. Winslow is inclined to the opinion, that the existence of any one predominant delusion, and that delusion not in the slightest degree associated with the criminal act, should be occasionally considered as a sufficient exculpation in criminal cases. The author, however, expresses himself so cautiously on this point, that we have but little difficulty in agreeing with him. The mere fact that a delusion exists in the mind is sufficient to create a suspicion of general mental perversion, and therefore to make out a primâ facie case of irresponsibility. Nor would we rest the exculpation of the accused entirely on the connexion between the delusion and the act being established by the evidence ; because 1, this connexion may exist but will not admit of proof; and 2, it may not exist, and yet there may be such a degree of mental disorder as to justify a plea of irresponsibility. It is, however, a very different matter to assert that the mere proof of a delusion existing or having existed in the mind should render a party in all cases irresponsible for his act. It may be a strong ground for suspecting insanity, but it is nothing more; we should not be justified in inferring irresponsibility until all the circumstances of the case had been fully investigated. It might turn out, notwithstanding the existence of delusion, that the crime had every mark of sanity about it, just as we find in the execution of wills and deeds that their validity is occasionally admitted in spite of the obvious existence of delusion.

The reasonableness of the act and the mode in which it is performed are in all such cases closely scrutinized; and a civil act is not necessarily held to be invalid, unless it bear about it obvious marks of the individual having been influenced by his delusion in drawing it up.

Mr. Winslow mentions some facts which prove beyond all doubt that persons confined as lunatics are capable of committing offences with the full knowledge that they would escape punishment by insanity being pleaded for them; in short, they are aware of the effect of the plea, a state of mind which some medical writers have held to be a well-marked criterion between one who is an impostor and another who is homicidally mad. It is certainly a fair matter for consideration, whether, when the mind is thus capable of drawing a perfectly sane inference as to the consequence, the individual should not be held as much responsible for his act as if no delusion were proved to exist. This of course involves the question of how far punishment would be attended with a good effect, a subject which we shall reserve for consideration hereafter. As far as the law is concerned, it is pretty certain that where so much knowledge of the consequences of an act was displayed, the individual perpetrating it would be held responsible. A man would not be allowed to forge a will or a deed, and escape upon the plea that being a lunatic, or labouring under some delusion, he should avoid the punishment due to his act, when, in the very perpetration of it, he contemplated an acquittal on the ground of insanity. If a man has sufficient knowledge to calculate upon an escape from punishment, it is reasonable to suppose that he has a sufficient power of self-control to prevent him from perpetrating the crime for which such punishment is commonly inflicted.

At p. 17 the author quotes a very instructive case, in which, as he observes, if the murder perpetrated had occurred *out of a madhouse*, with all its attendant circumstances, there would have been no doubt as to the justness of the penalty had the individual been condemned to expiate his crime on the gallows. A man confined in a lunatic asylum had been subjected to very cruel treatment, and he killed the person who had the care of him. For the act itself there was the strong motive for revenge; the man had threatened the life of his keeper; and in the perpetration of the murder, there were displayed cool premeditation, precaution, and concealment of the means, which are commonly considered as characteristic of the sane assassin. The man, known to be insane, was justly acquitted; but had he been at large when the act was committed, it might be questionable whether his execution might not have been justifiable. A mind which was thus able to display all the usual marks of sanity in the perpetration of a heinous crime, would be influenced by the example of punishment; and others, like this man, might be thereby so deterred from the perpetration of murder under similar circumstances. Unless some such rule as this be adopted, it is difficult to understand how we should ever be justified in inflicting punishment for any offence. Exculpation cannot depend merely upon the fact of a man being confined in an asylum; for, while a responsible sane person may be thus unjustly confined, another who might with propriety be considered irresponsible may be enjoying his liberty.

The case of Earl Ferrers, who was executed for the murder of his steward about the middle of the last century, has given rise to great difference of opinion among jurists and physicians. Lord Erskine thought that the Earl was properly executed for the crime of murder; because his resentment was not founded on any illusion (delusion ?); but it depended upon *actual circumstances and real facts.* Dr. A. Combe considers that the Earl was unquestionably of unsound mind, because he had been long beset with unfounded suspicions of plots and conspiracies, unconnected ravings, sudden starts of fury, and a strange caprice of temper; it was proved that insanity was an hereditary disease in the family, that several of his relatives had suffered from madness, and at one time his nearest relations had actually debated on the expediency of taking out a commission of lunacy against him. Mr. Winslow does not concur in this view; because the quarrel which the Earl had with the deceased did not originate "in any morbid images which had fastened themselves on his imagination, but it was founded upon existing facts." (p. 27.)

It appears to us that Mr. Winslow is here guilty of some inconsistency. The very strong presumptions in favour of the existence of insanity in Lord Ferrers's case weigh as nothing in his judgment; the question of responsibility is here made to rest upon whether the act was or was not founded on existing facts. We do not exactly perceive how he can reconcile this opinion with the conclusion to which he comes respecting the lunatic who murdered his keeper, and who was acquitted because he happened to be the inmate of an asylum. "We know so little of the workings of the human mind, either in its healthy or morbid state, that it is a point of great difficulty, in fact almost an impossibility, to detect the line of demarcation between responsibility or irresponsibility, or where one commences and the other terminates." (p. 18.) We do not see why this rule should not be equally applied to the case of Earl Ferrers: the lunatic had the same motive—revenge, and the murder was premeditated; in short, the crime was "founded upon existing facts." The lunatic had been ill treated, and knew of no other way of punishing his keeper than by killing him. It is a question whether the violent resentment which Lord Ferrers had imbibed against his steward, without a sufficiently reasonable cause, was not in itself a strong proof of his mind being disordered. We cannot, therefore, understand how in the one case the crime is to be referred to passion and in the other to insanity. We consider this test of looking for "existing facts" as decidedly fallacious. Lunatics are certainly capable of committing crimes under the influence of what may be called sane motives, as for example from revenge or passionate excitement; the quarrel preceding the crime may not have originated in any morbid images, but have been founded upon existing facts; and if this is to be made the test of responsibility, many would be condemned to suffer whom the very advocates of the test would pronounce to be irresponsible for their acts. The recent decisions in the cases of Oxford and M'Naughten, contrasted with those of Lord Ferrers and Bellingham, attest the uncertainty thrown around the medical jurisprudence of insanity. In the two former cases the proofs of insanity in the general conduct of the parties were feeble, compared with those which existed in the latter; and it is impossible to come to any other conclusion than that if Oxford and M'Naughten were properly acquitted on the ground of insanity, Lord Ferrers and Bellingham were most unjustly convicted. We shall reserve any further remarks on this subject until we have occasion to examine the evidence given on the trial of M'Naughten.

On the subject of moral insanity, we do not find anything that is new in Mr. Winslow's book: this part of the essay is mostly made up from cases and observations extracted from the works of well-known authors. The same may be said of the section on homicidal insanity; the author thinks that "in the majority of these cases the intellectual faculties, as contradistinguished from the moral perceptions and powers, give no evidence of disease," (p. 60,) although he is inclined to believe that these horrible destructive impulses will generally be found, when the case is properly examined, to be connected with some hallucination or perversion of the mental faculties.

The characters which are supposed to distinguish the homicidal monomaniac from the sane criminal are quoted from the work of Dr. Prichard. Upon these we have little further to remark. We admit that in homicidal monomania we commonly find the crime to have been preceded by certain peculiarities of conduct; sometimes by a total change of character in the perpetrator, and there have often been previous attempts at suicide. These supposed criteria have, however, been repeatedly and very properly rejected, when tendered as evidence of insanity in courts of law. They are of too vague a nature, and apply as much to cases of moral depravity as of actual insanity; in short, if these were admitted as proofs, they would serve as a convenient shelter from punishment for most criminals. The third point is absence of motive, upon which some remarks have already been made in our notice of Dr. Prichard's work. It appears to us that Mr. Winslow is more inclined to adopt the opinions of others than to examine them with the care which the importance of this question demands. He must be aware that if, before inquiring into the perpetration of a crime, we were to search for the motives, and rest the responsibility of an accused upon the accidental discovery of what we might deem reasonable motives, many most atrocious criminals would necessarily go unpunished. We have already adverted to the case of Courvoisier; but this is not a solitary instance of the extreme fallibility of such a criterion. In the atrocious murders and mutilations perpetrated by Greenacre and Good within the last few years, we have additional proofs of the utter futility of trusting to this test for homicidal monomania. No reasonable motive was ever discovered in either of these instances; and yet the parties were very properly made responsible for the crimes. We could here enumerate many similar cases; but we have elsewhere sufficiently stated our views of this subject. The absence or rather the non-discovery of a motive for a crime like murder, can only be admitted, in support of a plea of irresponsibility, when the existence of insanity is clearly established by other facts. Unfortunately, however, medical witnesses rely upon this as one of the strongest proofs of insanity; although cases are continually occurring which show that many atrocious offences are perpetrated without any *apparent* motive. It is not the office of a human tribunal to search into motives; this is a matter which lies between man and his Maker; and it is obvious that if this were considered a necessary preliminary proof on trials, and the accused were to be convicted or acquitted according to whether a motive for a crime was or was not discovered, it would be a complete sacrifice of the safety of society to false metaphysical doctrines. Another character assigned as a ground of distinction is, that the murderer has generally accomplices in vice and crime. It must be apparent, however, that this is far from true; some of the most atrocious murders committed in modern times have been the acts of solitary individuals, who had neither accomplices nor any assignable inducements leading to the perpetration of the crimes. The cases of Courvoisier, Greenacre, Good, Bolam, and Cook, the murderer of Mr. Paas, sufficiently bear out this statement; and in the absence of motive, of accomplices, and the atrocious nature of the crimes themselves, these cases certainly fall under the usual definition of homicidal monomania. But the criminals were properly held responsible and were punished; a clear proof of the insufficiency of such arbitrary rules, to enable us to distinguish between the homicidal monomaniac and the sane murderer.

There is one other criterion which carries with it some force: "The subsequent conduct of the unfortunate individual is generally characteristic of his state. He seeks no escape or flight, delivers himself up to justice, acknowledges the crime laid to his charge, describes the state of mind which led to its perpetration; or he remains stupefied and overcome by a horrible consciousness of having been the agent in an atrocious deed." From an examination of many cases of homicidal monomania, we are bound to state that this is a character which is almost uniformly met with. The monomaniac commits the crime openly, and when charged with it, he neither attempts to deny or to conceal it. By the sane criminal, however, every attempt is made to conceal all traces of the crime, and he denies it to the last; examples of which are furnished to us in the cases of Courvoisier, Greenacre, Bolam, and others. But let us not be misunderstood; we would not say that this should be made a test of homicidal monomania. Persons who destroy the lives of others through revenge or anger, often perpetrate murder openly, and do not attempt to conceal the crime when they know that concealment is hopeless. Besides, a morbid love of notoriety will often induce sane criminals to attempt to commit assassination under circumstances where the attempt must be witnessed by hundreds, and there can be no possibility of escape. The English law has, however, more than once rejected this as a test; and proceeded to punish the individual in the same way as if he had attempted secret assassination, and had endeavoured to conceal his attempt afterwards. We may remind our readers that the attempt made by Francis on the life of the sovereign was followed by conviction and punishment, although it was publicly made and the assassin did not attempt to escape. Indeed, it is obvious that the notoriety attending the offence would be the only conceivable inducement to its perpetration. What possible motive, we would ask, could ever exist in the mind of a subject for attempting to assassinate the sovereign of this country? And if, according to Dr. Prichard and Mr. Winslow, we are to take the absence of motive, the want of accomplices, and the fact of a man attempting assassination openly, and using no effort to escape, as tests of homicidal monomania, and sufficient for a plea of irresponsibility, it is clear that under no circumstances should the assassination of the sovereign be held to be a crime. It would be better at once to declare that this detestable act of treason should be deemed sufficient evidence of insanity,—that the perpetrator should be regarded as "an unfortunate" person, and exempted from all responsibility for his act. We do not say that these authors would adopt this extreme view, but their arguments lead directly to its adoption. It is sufficient for us to state, that this revolting doctrine has been negatived by the law in the case of Francis; and we believe that in this case the verdict met with the full concurrence of society. Notwithstanding the evidence of peculiarity of conduct in the accused, of the act being without motive, of its being perpetrated openly, of the individual seeking no escape or flight, but delivering himself up to justice, of there being no accomplices in vice or crime, the law has, and we think justly, pronounced that these facts did not exempt him from responsibility for the act. It therefore appears to us, that these alleged criteria of homicidal monomania must either be abandoned as insufficient and dangerous of application, or those who advocate them must be prepared to say, that atrocious crimes involving the safety and well-being of society may be perpetrated with comparative impunity.

It may be said :—if these are not to be received as characters of homicidal monomania, what other distinctions can be drawn? We reply that if we are not yet in a condition to draw a line of demarcation between responsibility and irresponsibility, it is something to have shown that the rules at present advocated by certain authors are insufficient, and liable to become dangerous in their unrestricted application.

With some of Mr. Winslow's sentiments we fully agree. Thus when he says, "There is no doubt of the occurrence of this form of insanity (homicidal monomania), and when its presence is clearly established the person so unhappily afflicted ought not to be considered as a responsible agent," (p. 67,) he states no more than would be immediately assented to by most persons who have reflected on this important subject. We do not, however, find that he has furnished us with any tests by which its presence can be *clearly* established. He has relied too much upon the vague rules given by others, without sufficiently examining their tendency or actual value.

Again; he justly complains of the test applied by the law to these cases, namely, whether the person committing the crime could at the time distinguish between right and wrong, good and evil—terms which, as we have elsewhere said, only tend to confuse the minds of juries. He says, "A person may be perfectly competent to draw a correct distinction between right and wrong, and yet labour under a form of insanity, which ought unquestionably to protect him from legal or moral responsibility." (p. 74.) This is undoubtedly true; and we think that it is fully borne out by the cases of Martin, Hadfield, and Greensmith. (No. XIX. p. 144.) The knowledge of the legality or illegality of an act at the time it is perpetrated is not always a safe criterion; and the only apology that can be offered for its adoption is this: that it is perhaps the sole criterion by which the conduct of a criminal can be tested before legal tribunals, as they are at present constituted, and that the test is never insisted upon by our law authorities where other facts plainly tend to show the insanity of the accused. Thus, then, it is not true that the test is rigidly applied or that "the unfortunate maniac has no chance of escaping." The cases of Martin, Hadfield, and others plainly refute this notion. The author must be aware that no direct rule of criminal law can be so framed as to meet all cases of supposed insanity; and that with an erroneous test substantial justice may be done, if the rule be relaxed in those instances where other circumstances would clearly render its application harsh. It is an essential feature of the *sane* criminal that he always shows by his conduct a knowledge of the *illegality* of the act which he is perpetrating; it is this which leads him to the adoption of secret and tortuous means of destruction, and also to the subsequent concealment of the crime. Thus, then, this rule, although it falls short of what may be required, is perhaps as well fitted as any other to guide the uninstructed minds of a jury.

The following extract is replete with good sense, and we here quote it, as it conveys an important caution to medical witnesses:

"He (a medical witness) should, when called upon to give evidence in cases of insanity, never forget that he has nothing to do with the legal definition of the term. No medical man is competent in every case to say whether the party supposed to be deranged is or is not competent to draw a line of distinction between good and evil, right and wrong. The legitimate point which the medical witness has to decide is this: Had the alleged lunatic, at the time he committed the offence, sufficient control over his actions? It is absurd to believe that any amount of medical information or metaphysical knowledge, which the witness may be in possession of, will enable him to form anything like an accurate notion of the lunatic's capability of distinguishing between right and wrong. Above all things, the medical man should avoid defining insanity. Counsel knowing the obscurity of the subject, and conscious of the difficulties with which medical men have to contend in arriving at a correct opinion, most unfairly, in their examinations, endeavour to tie them down to definitions; and then by showing their fallacy weaken the whole effect of their testimony. There is nothing so easily seized upon as a definition, therefore the witness should be cautious in committing himself by attempting to define insanity. It will be better and wiser for him at once to acknowledge his incapacity to do so, than by a vain and ostentatious display of metaphysical lore to peril the life of a fellow-creature." (p. 76.)

In admitting the justice of these remarks, it must not be forgotten that there are two sides to this question; and that as yet more "metaphysical lore" has been displayed in endeavouring to prove criminals insane than in establishing sanity on the part of criminal lunatics. It is only fair to the author to state, that he entirely disconnects himself from those who think that the presence of a disordered mind ought invariably to shield a person from responsibility. The degree and character of the mental derangement should be looked to; the existence of waywardness of character, idle fancies, and absurd delusions, should not of themselves screen a person from the penalty awarded by the laws for a criminal offence. The doctrine that such persons should be exonerated from all responsibility is, in his view, as unphilosophical as it is opposed to the safety of society.

In dismissing Mr. Winslow's book, we have to remark, that in our judgment the subject might in a future edition be better arranged. The treatise would advantageously admit of a division into chapters, and some parts of the subject require to be more fully treated. In other respects, there is much in the work to suggest matter for deep reflection to those engaged in inquiries of this nature.

III. We have now to examine the work of Mr. Sampson, in which we find an entirely new view taken of the medical jurisprudence of insanity. The great object of the author in this treatise appears to be to establish three points: 1, that the test of insanity is obedience to the laws; 2, that the commission of crime is ipso facto a proof of insanity; 3, that capital punishment, whether for treason or murder, is barbarous, inexpedient, and unjust. With regard to the first point the author remarks:

"Although it cannot be maintained that there exists any human mind in a state of perfection, yet we may consider, as perfect for all social purposes, that mind which comes up to the average state (?) of mental power characterizing the society of which it is a part. This average state of the social mind is precisely indicated by the laws and institutions which society frames or permits to be framed for its own governance; and hence it may be very safely taken as a rule, that every person is sane to the requisite extent for the performance of social duties, so long as he possesses the mental power and disposition to act in obedience to the laws." (p. 6.)

This is what we presume may be called social sanity. As the laws of countries differ widely from each other, what is sanity in one may, upon Mr. Sampson's doctrine, be insanity in another. But there are two classes of persons for which this singular definition of sanity does not provide; namely, 1st, those who infringe the laws from ignorance, and who can hardly, therefore, be pronounced insane; and 2d, those who having been born and bred under one system of laws, find themselves in after life in another country, where the customs, of which they may be utterly ignorant, are different. It may be said obedience to the law implies a knowledge of it; we reply that such a doctrine is practically disregarded: if ignorance of the law were a sufficient answer to any criminal charge, few convictions would take place. In some eastern countries, murder is scarcely regarded as a crime; but the English law would not allow an inhabitant of these countries, who had committed murder in England, to escape, merely because he had just arrived and was ignorant of the fact, that what he had been taught to look upon as a venial offence in one place was a capital crime in another. For this reason, we do not think the rule proposed by Mr. Sampson either safe for the individual or for the society of which he is a member. If it be urged that a man might possess the mental power and disposition to act in obedience to the laws, provided he knew them, the definition appears to us to be frittered away. His sanity would then be a continually fluctuating quantity, varying with the care and attention which he had bestowed upon the subject of legislation, and with the kind of society of which, for the time, he formed a part.

The second proposition appears before us in the following form:

"We are bound, when a person has committed or attempted to commit a crime, to receive that fact as a sufficient evidence that his brain is in an unsound state, the degree of his unsoundness being indicated by the extent of his offence. The recognition of this fact necessarily leads us to the conclusion that the infliction of punishment in any case whatever is wholly inconsistent with all ideas of justice." (p. 24.)

Thus, then, there is one degree of insanity for larceny, a second for rape, a third for murder, and a fourth for treason. In every case the individual perpetrating the crime is to be regarded with pity, as an unfortunate sufferer :

"It would be quite as rational," says our author, "to flog a man at the cart's tail for having become infected with the scarlet-fever, owing to a predisposition and exposure to the disease, as to pursue the same course to one who, falling into temptation, had given way to a predisposition for taking possession of whatever he could lay his hands upon. To be sure, it might be said that the flogging could not operate so as to deter the man from catching another fever, while it might deter the thief from repeating his offence; but this distinction will not hold good, because, in the first instance, dread of the punishment might possibly induce the patient to attend in future so closely to the laws of health as to keep him safe from infection, (!) and it could do no more in the latter case with regard to the laws of morality." (p. 35.)

To the very obvious objection that in one case the mischief done affects only the person, while in the other it affects the security of society, the author replies by ingenious sophistical arguments. Thus, it is said, "society is liable to suffer even more seriously by the impairment of the bodily health of one of its members than from the results of the moral depravity of another." (p. 35.) The "moral offender" may, it is true, commit one or more murders, but this is balanced by the physical sufferer not being able to devote his energies to the good of society (!); and in addition to this, society may suffer far more seriously by the "man of ruined constitution transmitting to another generation his own delicate and enfeebled powers." (Ib.)

The author then proceeds to reason himself into the belief, that the infliction of punishment for a crime, which he assumes to be always indicative of disorder of the brain, is no more reconcilable to our ideas of justice, than the infliction of punishment on a man because he is labouring under inflammation of the liver or lungs. We are not surprised that he should himself be somewhat startled at the conclusions to which these singular doctrines lead. He admits, as one objection, that they would tend to destroy all ideas of responsibility; in which we perfectly agree with him. It is true, he attempts to show that this objection is without any solid foundation; but we are by no means satisfied with the explanation which he offers; it is a tissue of ingenious special pleading, and nothing more. All persons, he says, are responsible for the acts which they commit; but this responsibility consists in making them undergo "the painful but benevolent treatment requisite for their cure."

Thus a man vicious and depraved may walk about the streets of this metropolis, and so long as he commits no offence, he receives no pity for his situation; he is even allowed to perish from want. If he commits murder, rape, or arson, and more especially if the crime be accompanied by any features of peculiar atrocity, he at once becomes, on Mr. Sampson's principles, a proper object of benevolent treatment: he may have committed a cold-blooded murder to possess himself of valuable property, rape from sheer licentiousness, and arson from revenge; still he is an object of pity: he could no more help perpetrating these crimes than he could avoid an attack of inflammation of the liver. Most medico-legal writers have thought that the existence of motives for crime should be at least looked for, and that there was a distinction to be drawn between cases where the murder was committed for gain and the crime concealed, and other cases where the killing took place under a fit of delirium or mania; but Mr. Sampson does not trouble himself with any such distinctions. All crime proceeds from insanity. Insanity and moral depravity are synonymous terms; thus, then, on this principle, one of two inferences must follow, either that all persons guilty of criminal offences should be punished, whether or not they be insane, in the common acceptation of the term, or that no punishment should be inflicted on a person for the perpetration of any crime whatever. "*Punishment from man is not necessary.*" (p. 55.)

Many eminent judges, among others Lord Hale, have argued that all crime was the offspring of partial insanity; but no one before Mr. Sampson has ever attempted to draw from this doctrine the inference that every criminal should be exonerated on that ground. There is no doubt that in most criminals there is a degree of moral depravity which prevents them from reasoning or acting like men of a healthy moral standard; but experience has long ago settled that a punishment proportioned to the offence has had considerable influence in checking the conduct of these depraved characters; and whatever ingenuity may be displayed in building up plausible psychological theories, there is a general feeling in society that the dread of punishment has considerable influence in restraining these wicked propensities of the vicious. Mr. Sampson may not agree with this view; but the common sense of mankind, and the most common experience of nations, are against him. The truth appears to us to be, that not finding himself able to draw a clear distinction between insanity and crime, he has thought it better to take up an extreme view at once: it is thus we find him involving himself in numerous inconsistencies.

We do not feel ourselves called upon to enter at length into an examination of the author's views with respect to punishment. The mere infliction of punishment is, in his view, an act of inherent and barbarous injustice; and he appears to regard capital punishment in the light of judicial murder, however atrocious the crime for which it may be inflicted. The substitute which he would propose is restraint, perpetual in the case of murder; because, although the homicidal tendency may be apparently removed, we never can be certain that the impulse may not again arise under the sudden influence of external excitement. During this time the individual should undergo treatment to remove the moral disorder, so to speak, under which he is labouring. "In lesser crimes, the same necessity for *perpetual* restraint does not exist; and therefore the period of the incarceration of the criminal should be contingent entirely upon his own improvement, and certainly need rarely be so prolonged as to terminate only with his life." The shallowness of this mode of reasoning scarcely requires to be exposed. The same motive which would justify perpetual imprisonment in cases of murder, would justify it in cases of rape, burglary, arson, and highway robbery; for how are we ever to predicate that the impulse to these crimes, destructive as they are to the peace and well-being of society, may not again arise "under the sudden influence of external excitement?" So of theft, or of any other crime; in short, it appears to us that this doctrine, carried to its legitimate extent, would condemn all offenders against the law, whether the traitor, the murderer, or the petty thief, to perpetual imprisonment. We have been taught to look upon this as a severe punishment, and most persons would so regard it: some have held perpetual imprisonment to be a more severe punishment than death itself; and yet, while Mr. Sampson advocates it openly for all cases of murder, and impliedly for other crimes, he contends that the infliction of punishment is an act of inherent and barbarous injustice. His reasoning stands thus: Punishment from man is not necessary. Perpetual imprisonment is necessary; ergo, perpetual imprisonment is no punishment.

Among the fallacious arguments adduced in support of the abolition of the punishment of death, we find it urged that capital punishment tends to keep up a blood-thirsty propensity among nations and individuals. The author says that in all countries where capital punishments are rare, the tendencies of the people are always proportionably humane. We deny this; and appeal to Italy and Spain, if he has any personal knowledge of those countries, as affording examples directly contradicting his statement. It is asserted that out of 167 persons executed during a certain period, 164 had been present at executions. The inference is that public executions tend to increase the number of murders. (p. 105.) Here we have another example of the saying that figures may be made to prove anything. Criminals are commonly found among the most depraved; and it is of the depraved members of society of which these crowds at public executions are chiefly composed; but the inference drawn is wholly false; for where probably one present at an execution subsequently commits murder, two hundred may not: hence, to judge of the effect of executions in the way suggested, we should know what proportion of the whole numbers present the 164 executed criminals formed. By this convenient application of figures, the author might equally prove that horse-races and fairs had an equal tendency to increase the crime of murder; for a large proportion of those who are executed would probably be found to be regular attendants at such places.

Then we have the old argument of the inefficacy of these punishments as examples, because persons who have witnessed them have soon afterwards committed murder. A little reflection will prove that this is an argument against any punishment at all. Persons just discharged from prison have been known to be apprehended within an hour for a repetition of the offence. The truth is, with some persons punishment of whatever kind entirely fails as an example; but there is good reason to believe that it influences the majority: so that solitary examples of this kind prove nothing.

The author has taken up the extraordinary notion that the punishment of death increases the number of murders, because some who wish to die commit murder in order to be put to death by the public executioner; and he does not hesitate to express his belief, "that a remarkable diminution in the number of murders by which our country is annually disgraced, would be the immediate consequence of its abolition." (p. 98.) Admitting, as we do, the fact upon which this assumption is built, we must remind Mr. Sampson, that many persons annually steal or commit other crimes in order that they may be transported; therefore, it may be argued, transportation encourages theft. So, again, offenders are continually brought before our police-courts who admit that they have broken lamps, windows, and committed other wilful damage to property, in order that they might be sent to prison; therefore, the abolition of prisons would be advisable; since there might be a great diminution in the number of offences of this description. Notwithstanding these well-known facts, it is considered that transportation and imprisonment tend to check crime; and if capital punishment does not check the crime of murder in a like degree, it is not for the reasons assigned by Mr. Sampson.

All right-thinking men agree that capital punishment was too frequently resorted to formerly, and wholly failed in its effect as example; but it is a general opinion, except with theorists like Mr. Sampson, that it would be unwise to abolish it in cases of murder. The laws of all civilized countries sanction it; it is considered to be the best security which can be afforded to society, and one that must be occasionally resorted to. Even those who, like the author, have recommended the substitution of perpetual imprisonment, have not said how a criminal is to be dealt with who refuses to submit to this punishment, who murders his keeper, who escapes from prison, and commits other murders or atrocious crimes upon innocent members of society. Mr. Sampson might consider such a man, although not a lunatic in the general acceptation of the term, as an object for increased benevolent treatment; but we apprehend that society would consider itself fully justified in putting an end to the career of a monster of this description, just as a man would in defending his life against the attack of an assassin.

Our readers will judge from the preceding remarks what our opinion is of this book. The doctrines are as pernicious as the reasoning upon which they are based is fallacious. The author blames one half of mankind for the immorality or depravity of the other; and while looking forward to a "Utopia," in which there will be no tendency to the perpetration of crime, he allows that the innocent members of society are to be openly the subjects of assassination and robbery, with no other punishment for the criminals than restraint and benevolent treatment. We can scarcely bring ourselves to believe that any person should in the present day seriously propound such wild and reckless views.

IV. We shall here briefly advert to the Commentary on Insanity by Sir A. Crichton, because it bears strongly on the subject which we have been discussing, and furnishes an answer to the crude doctrines of criminal responsibility which we have considered it our duty to expose. The definition and treatment of insanity require no remark. The author objects to Dr. Prichard's definition of moral insanity, as comprehending "a number of diseased affections as well as varieties of moral character which have no similitude to each other, or even to true insanity." (p. 186.) This is undoubtedly correct; Dr. Prichard must himself perceive that his "moral insanity" may be easily made to include all cases of moral depravity; for we have elsewhere shown that the distinctions which he has attempted to draw between the two classes of cases are artificial and often inapplicable.

Sir A. Crichton, like all other medico-legal writers, denies that crime is necessarily indicative of insanity, or that every criminal is insane. Perhaps he carries his views on this point a little too far, and is inclined to look upon many persons, who would probably be acquitted on the ground of insanity, as being responsible for their acts. Nevertheless, we highly approve the manner in which he discusses the subject.

"In our fallen state, evil propensities and bad passions are the inheritance of every descendant of Eve; but experience teaches us that there is not a propensity or passion to which we are subject which may not be weakened and rendered even inoperative by accustoming an individual to associate with its first impulse or suggestions emotions of a contrary nature. Hope and fear are the great engines. It is on this principle that moral education depends. It is in this way alone that vicious propensities in youth can be corrected. But it is too much to say, that because many unfortunate individuals have never been accustomed by due instruction and discipline to exercise their reason and to associate in their minds the dread of future or temporal punishment with evil propensities and sinful acts, therefore they ought to be considered as irresponsible agents and lunatics." (p. 190.)

The unsettled state of opinion which at present exists among medical men on the subject of insanity is, in his opinion, the source of two evils,—that some criminals who ought to be punished are acquitted of responsibility, while, in other instances, true homicidal monomaniacs are convicted and executed. It is difficult to say of which evil society has now the greater reason to complain; but it is a matter of sad reflection that the result depends less on the facts proved than on the medical and legal ability and ingenuity engaged in the prosecution or defence. The more conspicuous the crime, the greater are the exertions made, and the result is commonly successful: on the other hand, trials frequently take place at our assizes for offences, affording primâ facie evidence of insanity, in which the accused is either undefended or left to the chance defence of some counsel in court, and the result is a conviction.

Sir A. Crichton differs from Dr. Prichard in thinking that there is often a power of control on the part of a criminal, who might be laboring under what is termed moral insanity; and this, as our author properly suggests, and as we have elsewhere urged, should be the real test of responsibility. A man is caught in the act of attempting murder,—he finds his antagonist better armed than himself,—he attempts to kill him, falls on his knees imploring mercy, or endeavours to escape by running away. "Such a variety of motives, from which he chooses one according to circumstances, show evidently that his conduct is guided by understanding, though at the time he is under violent excitement." (p. 194.) This adaptation of mean to ends is certainly a strong proof of a reasoning power, and consequently of a power of self-control,—its existence may be frequently inferred from the manner in which the crime is perpetrated.

Medical men have, on these occasions, passed often beyond the legitimate sphere of their duties. It is not for a medical witness, as such, to discuss the subject of free-will, human responsibility, or the degree of punishment which should follow an offence. By so doing he usurps the functions of the judge and jury, and takes upon himself to dictate to society the means which it should employ for the protection of its members. In these cases, his professional studies give him no advantage over the common sense and experience of mankind,—a fact made evident by the very different opinions entertained on these subjects among equally eminent members of the profession. A medical witness has only to state to what degree, in his opinion, the mind of a person accused of crime deviates from the ordinary standard ; while, from the evidence given on some recent occasions, it would rather appear that the accused was tried by the medical witnesses than by the court before which he was arraigned. On this point Sir A. Crichton very justly observes :

"If revealed truth, which declares that we are to answer for the deeds we commit in the flesh, is discredited, it is not by metaphysical arguments that we can hope to settle our faith; but this every one must needs know with certainty that in this country, and in every other civilized state which is careful of the interests of society, the laws are founded on the doctrine of the freedom of the will, as it is called, or on human responsibility ; and also on the belief, that examples of punishment and the dread of future condemnation will influence the determinations and actions of men until they are bereft of reason by real disease. To the judge must be left the task of considering the circumstances which palliate offences, and make criminals objects of special commiseration and mercy; but the attempt on the part of learned doctors in law and medicine to confound vice with insanity, and, consequently, to condemn the right of human punishment, I consider as one of the many dangerous innovations which the proud philosophy of the nineteenth century has produced." (p. 195.)

IV. The last three pamphlets on our list refer to the late trial of M'Naughten for the murder of Mr. Drummond. The first of these is a non-professional report of the trial, with the whole of the legal arguments and medical evidence, unaccompanied by comments. It should be in the possession of all who feel an interest in the medical jurisprudence of insanity ; for probably there is no case in modern times which has excited so much attention, in and out of the profession, as this.

We consider it unnecessary to detail the facts of this case : they are so recent that they must be familiar to the whole of our readers. We shall here offer only a few remarks on the defence : this was to the effect that at the time the accused perpetrated the act he was laboring under homicidal monomania. It was deposed to by many witnesses that the prisoner was latterly of a sullen and reserved character; that he imagined himself to be the object and victim of the most unrelenting persecution ; that he was surrounded by persons who had formed a conspiracy against his comforts, his character, and even his life; and that wheresoever he went these persons still pursued him, and gave him no rest either by day or by night. It also appeared that he imagined the deceased, who was a perfect stranger to him, to be one of his persecutors, and that it was necessary he should fall a sacrifice in order to free him from persecution. There was no proof of intellectual insanity about him, if we except the existence of these delusions ; and it was admitted by all that he was shrewd in business transactions, that he was fully competent to the management of his affairs, and had realized a considerable sum of money by his own industry in trade.

These were the principal points in the defence; the remainder of the evidence in favour of insanity being made up by the opinions expressed by the medical witnesses. We will now compare this evidence with those characters which have been assigned by Prichard and others as proofs of homicidal monomania. We have already expressed our opinion that these characters are loose and vague; but the counsel for the defence chiefly based his arguments in favour of the prisoner's insanity upon them. There had been certain peculiarities of conduct and absurd delusions; the man was of a morose and reserved disposition, but we do not collect from the evidence that he had ever attempted suicide. It does not appear that he was ferocious or cruel: his counsel dwelt much on his humanity to the brute creation, a fact which, in his view, did not accord with the ferocity of a sane assassin. (p. 46.) In this we have a good example of legal ingenuity; for one strong feature of moral insanity, leading to homicidal madness, is cruelty or ferocity of disposition; so that some physicians have given to it the name of brutality. Thus it will be seen that the very reverse of the usual condition was received without comment as a proof of the existence of homicidal madness. In the language of Mr. Rumball, there was no indication of diseased destructiveness about him. We put no great stress upon this, one way or the other, or upon the absence of the suicidal tendency before and after the crime; but it shows that the proofs of homicidal insanity are of so ambiguous a character that a barrister may select either of two opposite conditions in favour of his argument.

Next we come to *motive*. There was no apparent motive on the part of the prisoner in shooting Mr. Drummond; but we think we have said sufficient to show that this should not be received as evidence of insanity; it merely makes out a primâ facie case for inquiry. Further, it was argued by the counsel for the defence:

"The manner in which the murderer sets himself to the consummation of his crime, as well as his subsequent conduct, is very different from the proceedings of a madman. The former often has accomplices; he commences with premeditation, lays a plan beforehand, chooses time, place, and circumstances adapted to the perpetration of the deed, and generally has contrived some method of escape. He always studies concealment and personal safety, and when there is danger of detection, uses all possible dispatch to escape the punishment due to his crime. All these particulars are reversed in the proceedings of the madman. ............ A common murderer would have acted in a different manner, he would have chosen a different time, a different place, he would have sought safety by escape." (p. 58.)

It is with something like dread that we witness these displays of forensic eloquence and ingenuity in questions of criminal responsibility. While, on the one hand, they may lead to the acquittal of one who is responsible; on the other, they may bring about the condemnation of an irresponsible agent: this is a pure matter of accident, depending on the fact of whether the ability be displayed on the side of the prosecution or defence. In the quotations which we have above made from Mr. Cockburn's speech for the prisoner, we have what appears to be well-marked points of difference laid down; although he had just before adduced and commented in favour of his views upon cases which completely overturn the differences thus sought to be established! Thus Hadfield's case is quoted among the instances of homicidal insanity; but probably there never was an attempt made upon life, in which there was greater premeditation, precaution, or a better choice of time, place, and circumstances, than in the attack made by this monomaniac on the life of George the Third! We also think it is obvious that if a man, whether sane or insane, have the design to shoot the sovereign, a minister of state, or any great public character, he can seldom have an opportunity of doing this except in public, and therefore under circumstances in which any attempt at escape would be commonly futile. With respect to accomplices, it is true that we never find them in cases of homicidal monomania but they are also generally wanting in crimes of peculiar atrocity and magnitude. The cases of Greenacre, Good, Courvoisier, and others, afford a sufficient proof of this fact. M'Naughten made no attempt to escape, or to deny that he had shot the deceased : this, as we have already observed, is a pretty uniform character of homicidal monomania; although a question might have arisen as to what he would have done, supposing he had not been seized in the act of discharging a second pistol. Still, however, allowing the prisoner the full benefit of this point in his favour, we must protest against this being drawn, as it was in this instance, into an absolute proof of homicidal monomania.

Mr. Cockburn adopts Lord Erskine's opinion of the case of Earl Ferrers, and considers, in opposition to the able commentary of Dr. A. Combe, that this nobleman was properly convicted of murder; although the evidence in favour of previous insanity was undoubtedly much stronger in this than in M'Naughten's case. The Earl did not attempt to deny that he had shot the deceased, and exclaimed when the act was done, "I am glad I have done it, he was a villain, and I am revenged." Yet, by the exercise of legal ingenuity, the same facts are in one case adduced as strong proofs of insanity and in another of perfect sanity. We have no doubt that had Lord Ferrers been as ably defended as some modern monomaniacs, he would have been acquitted on the ground of insanity; and if we compare the verdict in his case with later decisions, his execution can be regarded in no other light than as a judicial murder. In the mean time, the facts in Lord Ferrers's case are made to serve two purposes: they furnish proofs in favour of responsibility or irresponsibility, according to the option of the advocate.

It is well known, that when murder is committed through the motives of passion or revenge, whether apparent or concealed, there is frequently no attempt made to conceal or deny the crime, there is no attempt at escape, and yet such persons are made responsible for their acts.

The only other point in the legal defence upon which we have to offer a remark is this, that the counsel adopted Lord Erskine's doctrine, i. e. in order that there should be irresponsibility, two facts must be proved: 1, that there should be delusion; 2, that the act of homicide should be connected with the delusion. Admitting that the first point was proved in M'Naughten's case, we do not see how in any part of the defence the delusion was brought to bear upon the deceased as an individual. It was urged that the prisoner considered him to be one of his persecutors; but he was a perfect stranger to him, and therefore the prisoner might as well have shot any other person in the queen's dominions; for it was of course a matter of accident as to who might appear to him to be his persecutors. From the decision in M'Naughten's case, then, we infer that there may be the most broad and unrestricted application of this principle relative to the connexion of the act of murder with the delusion.

Eight medical witnesses gave evidence on the prisoner's state of mind. They all agreed that at the time he committed the act he was labouring under a delusion, and that he was led on by an impulse so irresistible, that nothing but a physical impediment could have prevented him from committing it. (Dr. Hutchenson.) It is remarkable that questions were allowed to be put to the witnesses on this trial, which have seldom been permitted on former occasions. Thus some were asked whether they considered the prisoner responsible for his actions—a question which has been hitherto left to the jury to decide from the medical and other evidence adduced in the case. There are strong objections to this mode of examination, for it is like placing the issue of guilty or not guilty in the hands of the medical witnesses; and we attribute to this, the great public dissatisfaction expressed at the verdict in M'Naughten's case. So long as a prisoner, or those who act for him, are allowed to select the medical witnesses, who are to speak to his state of mind, we think it would be at least prudent not to permit questions to be put in this form. If the witnesses are really independent, and they might easily be made so for this purpose, there could be no objection to the prisoner having the benefit of their opinion under these circumstances. But it is obvious, that the counsel for a prisoner would never summon any witness who could not speak in his favour; and if what this witness is to deliver under the name of evidence substantially includes the verdict of the jury, we do not see why the prisoner and his friends should not be at once allowed to select their own jury. Let us suppose in a case that twenty medical witnesses are appealed to, and while one half agree that the case was one of insanity, the other half do not; it is very clear that only the ten first witnesses would be called for the defence; and, unless an equal amount of industry were displayed on the part of the prosecution, the verdict must be carried in the prisoner's favour. It will be impossible, we think, to eradicate the suspicion of unfair dealing on these occasions so long as this bad practice is adhered to. The result concerns the country, and if medical witnesses are in any case to assume the functions of the jury, their selection should lie with the country and not with the prisoner. In this case the evidence was felt to be so conclusive in favour of the existence of homicidal monomania, that the jury under the direction of the judge acquitted the prisoner on the ground of insanity.

The case of M'Naughten has called forth many comments, a strong impression having existed, both in and out of the profession, that the plea of insanity had been stretched to an improper extent. On this point most men will of course form their opinions according to their reading and experience on the subject; nor will these opinions be much influenced by what is said by journalists and reviewers. In accepting the verdict of the jury as the only verdict which the medical evidence would warrant, we are bound to state that in our judgment there is no case on record, if we except that of Oxford, where the facts in support of the plea of insanity were so slight; and when we reflect upon the cases of Bellingham, Lees, and Cooper, the last two tried at the same bar and executed within the last few years, we feel that there is both uncertainty and injustice in the operation of our criminal law. Either some individuals are improperly acquitted on the plea of insanity, or others are most unjustly executed.

This state of things requires to be remedied: it is wholly inconsistent with our views of justice, that the acquittal or conviction of supposed lunatics on capital charges should depend not on the merits of their respective cases, but on the ability and ingenuity of counsel, and the metaphysical speculations of medical witnesses upon the question of criminal responsibility. One case becomes a subject of prominent public interest, and every exertion is made to construe the most trivial points into evidence of insanity; an acquittal follows. Another case is left to itself; and, as the line of demarcation between sanity and insanity is scarcely appreciable without good legal and professional assistance, the accused is necessarily convicted, and either executed or otherwise punished; although the proofs of insanity, had they been as carefully sought for and brought out, would have been as strong in this as in the former instance. In point of fact, there is no more stability in judicial decisions than there is in medical evidence; and we think that one great improvement in the present system would be to leave the question as to the state of the mind only to the medical witnesses; and that of responsibility and punishment for the act, to the judge and jury.

M'Naughten's case has certainly proved that there is a very narrow line which separates crime from insanity; and the expressed intention on the part of certain members of the legislature to bring forward some preventive enactment shows that there is at least a well-founded dread that after the result of this trial, the plea of insanity may be carried too far. The verdict has completely overturned the old doctrine of implied malice in law; for, after this, how can a person be convicted of murder for killing one who is wholly unknown to him? If a man wilfully, and without provocation, fired a gun into the midst of a crowd and killed a person, this was formerly held to be murder, since the act was considered to imply malice against all mankind; but it might now be fairly contended, the individual was not responsible; "he had no accomplices, he had no motive for the act, he did not watch his opportunity and kill the person secretly, he laid no plan for his escape, and did not attempt to escape after perpetrating the crime; he made no denial of the crime, but calmly resigned himself to his fate." This is the substance of Mr. Cockburn's defence in M'Naughten's case; and, taking this as a precedent, such a defence would probably lead to an acquittal under the circumstances above supposed, on the ground of insanity. It might be contended that an act so committed would of itself always indicate insanity; we, however, would say that the act might sometimes depend on moral depravity, and that then the perpetrator should be dealt with differently to one who killed another in a fit of delirium, or during a paroxysm of mania.

V. Mr. Rumball's pamphlet is chiefly devoted to an examination of the case of M'Naughten. He says of it, "There is not a man in the country who does not feel that the late decision was a legal but not an equitable one,—that a foul murder has been done and justice is unsatisfied." (p. 11.) The author's views of insanity are strictly phrenological, and in our opinion are much more sound than those advocated by Mr. Sampson. He considers that "So long as a man can subject his delusions to the correction of his own understanding, he is not mad, but he is mad the moment the delusions refuse to be controlled. So long as his feelings, however intense, can be antagonized and conquered by other feelings, whether fear or affection, so long is he sane." (p. 13.) His opinion, therefore, is, that "Insanity is the excitement of any of the mental faculties beyond the control of the remainder." (p. 15.)

Mr. Rumball occupies some space on the question of the expediency of capital punishment in any case. He admits that, although the putting another to death is inherent as a right, it is not expedient; and he considers that if the punishment of death were abolished and imprisonment substituted, juries would not hesitate to convict on these occasions, and judges to sentence. We believe that this is the best argument which can be adduced for the abrogation of capital punishment, and that in many cases the plea of homicidal insanity would not have been raised, but for the fact that conviction would probably be followed by death. It appears to us, however, that Mr. Rumball loses himself in the argument, for those who are acquitted on the ground of insanity undergo the very punishment which he would substitute for death, namely, perpetual imprisonment: therefore we are positively at a loss to know, why he should complain of the acquittals of Oxford and M'Naughten. In respect to the latter, he says, " the assassin is suffered to escape," (p. 25;) but this is not true; the man is as much punished as if the author's own alteration of the law had taken place, and the judge had sentenced him to imprisonment. It is true that he is not imprisoned for having shot Mr. Drummond, but to prevent him from shooting others. Again, we find him asking the question, " Was Oxford labouring under a delusion terminating in homicidal climax? did he ever pretend to be so? was he or was he not perfectly aware, that wrongfully to kill was murder?" (p. 28.) We certainly never could comprehend why Oxford should have been acquitted and Francis convicted for an offence perpetrated under such similar circumstances; but we do not see that Mr. Rumball has any reason to complain, when Oxford is undergoing the punishment which he himself recommends for infliction in all similar cases.

We must here take leave to remark, that medical men venture entirely out of their province when they discuss the subject of punishments. The great object of all punishment is to prevent crime, not merely to put it out of the power of the same individual to repeat his offence against the laws, but to prevent other reckless and depraved persons, of whom there are numbers in society, from imitating him. This question, therefore, falls more within the department of the statesman and legislator than of the physician or surgeon. The studies or duties of the latter cannot possibly fit them for deciding on the effect of punishment.

There are certain atrocious crimes which it is considered should be punished with death, as this is the only punishment which our legislature holds to be fitted for their repression. Whether it answers this purpose or not cannot be determined by an individual who has not exclusively devoted himself to the study of the subject for many years, and who has not had ample opportunity of determining the general effects of punishment on mankind. We can hardly say that homicidal monomaniacs are acquitted: they are imprisoned for life, and the great discussion which has arisen on some recent decisions is really whether this punishment, for it must be so considered, will have any effect in deterring others from imitating the crime.

Perhaps, as Mr. Rumball suggests, it may have this effect much better than if they were executed as criminals. Upon that point we shall offer no opinion, since it can only be settled by experience, and we should decline debating a question of such importance with one whose views must be founded on mere theory. We have no doubt that many, for whom the plea of homicidal monomania would be raised, are to be deterred by the effects of punishment: some of them have sufficient reason to know, that what they are about to perpetrate is contrary to law, and that the act will certainly be followed by imprisonment, and probably by death, if the plea of insanity should fail. Thus, then, some preventive means are in force; and we consider that the great question of the expediency of continuing capital punishment, or of substituting for it perpetual imprisonment, is in their case undergoing a trial. Homicidal monomania is well known to be imitative; and it is fair to suppose that this tendency to imitate is a proof that all power of reasoning and reflection is not lost. Society is at least justified in endeavouring to coerce such impulses; and the knowledge that perpetual imprisonment will certainly follow the attempt may have an effect on the minds of some. Those who are labouring under violent mania or delirium cannot be so influenced as to their acts; but we consider that the cases of such men as Oxford are not to be placed in the same category with these.

VI. With respect to the pamphlet on the Amendment of the Law of Lunacy, we have no doubt the anonymous author means well; but he has clothed his opinions in obscure and somewhat flippant language. The letter is addressed to Lord Brougham, and is a violent attack upon the opinions of all who differ from the author on the nature of insanity. He does not seem to give any credit to others for entertaining conscientious views on the subject; but his inference is the common one of persons writing with more enthusiasm than discretion, namely, that he himself is right and every other individual wrong. It is to us a matter of surprise that so many ingenious writers on insanity, while endeavouring to expose the delusions of others, should invariably fall into delusions themselves.

There is one good suggestion made by the author in his letter, namely, that questions relative to insanity should be left to be decided by an English board of "experts" of competent knowledge, and appointed under proper restrictions, as to their independence. We must all agree that the practice of taking opinions on this subject from witnesses specially selected by those who are to benefit by their evidence, or from persons accidentally present at the proceedings, is radically bad, and should be forthwith abolished.

To this pamphlet are appended the speeches of Lords Lyndhurst and Brougham in the House of Lords on the case of M'Naughten; and in commenting upon these, we shall endeavour to see what medical or legal inferences can be drawn from the foregoing considerations on the question of criminal responsibility. We must repeat a complaint which we have elsewhere urged, (No. XIX. p. 140,) that the legal test adopted by our judges is, with due respect to these great authorities, imperfect. Lord Brougham doubts whether the juries before whom the question is tried really comprehend what is meant by it. Most judges, in laying down the law of responsibility, tell the jury that to make a man responsible, he must be capable of knowing *right* from *wrong* at the time of committing the act. This Lord Lyndhurst stated to be the general law of the land, and that any alteration in it was impracticable; Lord Brougham properly complained that the test was vague and unsatisfactory; and while one judge required a knowledge of right and wrong, another required a power of distiuguishing between good and evil, a third that a man must know what is proper or wicked. (p. 24.) Such nice differences as these should rather be addressed to a jury of metaphysicians, than to one composed of plain simple-minded men.

Lord Brougham suggested that the test ought to be whether a man knew that the act he was committing was legal or illegal; in other words, whether, as it is sometimes expressed, the prisoner was aware that his act was contrary to the law of God and man. Now the objection to every one of these tests is that they do not answer the purpose intended. A man may know that the act of murder or incendiarism which he is perpetrating is wrong, that it is an evil, wicked, and illegal act, and yet be a homicidal monomaniac. If the test thus laid down by our law-authorities be correct, and be, as Lord Lyndhurst pronounced it, the general law of the land, we would ask why were Martin, the incendiary, and Hadfield, the assassin, acquitted? The test may appear to answer in some cases: it is also undoubtedly convenient of application; but, if generally and rigorously carried out, it would certainly consign to death many monomaniacs; if it cannot be carried out, it must be erroneous, and some other should be substituted. The true and only test of responsibility appears to us to be, whether or not the individual had at the time *any power of control over his actions.* (See No. XIX. p. 140, and the case of Greensmith, p. 144.)

It has been seriously argued that some remedy is required to amend the present state of the law relative to insanity in criminal cases. This can only be applied, as Lord Lyndhurst remarked, by adopting some measures of precaution to prevent the recurrence of such evils; but it is difficult to understand how this can be done. Are we justly entitled to confine a man who displays habits of eccentricity and waywardness; who is sullen, morose, and reserved; who is ferocious in his disposition, or, on the contrary, remarkable for his humanity to the brute creation; who fancies himself persecuted, while he is shrewd in business, industrious in his habits, and shows no sign of intellectual aberration? We should say certainly not; and that any medical man, who, as the law now stands, signed a certificate for his confinement as a lunatic, would probably have to pay a heavy penalty for his indiscretion. The whole world would exclaim against such a person, on such grounds, being consigned to perpetual imprisonment among lunatics; and yet anything short of this must expose one or more innocent members of society to the risk of assassination. The memorable case of Anderdon *v.* Burrows has rendered medical practitioners scrupulously cautious in respect to the signing of certificates of insanity; and we doubt whether before the commission of their crimes any member of the profession would have felt himself justified in consigning to an asylum such men as Oxford and M'Naughten. Even where a person has indulged in wild and reckless conduct, has given way to vicious and depraved habits, threatened the lives of his parents and friends for no apparent motive, and has passed his time in gambling-houses and brothels, the law has determined that there was no ground for the imposition of restraint. A case occurred to our knowledge within the last few years, in which these facts were proved of a particular person; he was sent to an asylum under the certificate of two medical men; he indicted the parties for conspiracy and false imprisonment, including the keeper of the asylum, his parents, and a respectable solicitor, who had testified to his insane habits, and this man obtained a verdict in his favour! The conduct of the defendants was considered to involve a serious infringement of the liberty of the subject; and this person is now roaming about the metropolis! We again ask, what can be done with such cases in the present state of the law? It is not to be expected that medical men or magistrates should incur the risk of actions or indictments for false imprisonment; and until some indemnity is afforded to those who act bonâ fide on these occasions, assassinations may be perpetrated, and the preventive remedy applied only after the mischief is done.

But we cannot conceal from ourselves that there are cases of homicidal monomania which no law and no means of precaution can reach. We allude to those where the murderous act is committed from a sudden impulse by a person who had previously displayed no immoral conduct and no sort of intellectual aberration. Instances of this description have been unfortunately frequent of late years; we have elsewhere related one. (No. XIX. p. 144.) Other examples might be found in the cases of Nicholas Steinberg, who killed his wife and four children at Pentonville in Sept. 1834; of a man named Staninought, a respectable tradesman, who killed his son in 1835; of Lucas, who destroyed his three children in March 1842; of Jessup, who killed his wife in Oct. 1842; and of a man named Giles, who cut the throats of two of his infant children at Hoxton in January of the present year. All these were fearful examples of homicidal mania, in which there were no previous symptoms indicative of insanity, or any irregularity of conduct on the part of the homicides, to justify the least interference with their civil liberty. Now it is clear that society can no more protect itself against such cases as these than it can against the effects of the earthquake or the volcano. Their occurrence is purely accidental; but one remarkable feature is, that the murderous propensity in these unrecognizable cases is commonly directed against those who are closely connected with the homicides in blood, and to whom they are attached by the tenderest ties.

There is one way, however, in which the legislature might interfere. Some of these dreadful crimes have been perpetrated under the strong impressions produced by the sight of artificial representations of similar scenes of blood and murder. In the case of Staninought, the murderous propensity, which led him to kill his only son, was clearly traced to the effect produced on his mind by the exhibition of the scene, in which the assassin Fieschi was represented with his infernal machine, and figures of murdered corpses lying around, with all the horrible details of that atrocious act of treason. After the exhibition, the man had neither sleep nor rest from the dreadful vision which haunted him, and he murdered his son the same evening. To those who have studied the subjects of the human mind, and who are therefore acquainted with the influence of the power of imitation on certain intellects, such a result cannot be surprising. The public exhibition of these scenes of blood with their disgusting details in private houses and in public theatres is a disgrace to the metropolis. There is scarcely a murder of any atrocity which is not thus represented; and the real clothes or weapons of the murderer are eagerly sought after to make the exhibition more appalling and attractive. Such exhibitions are visited by thousands; and among these, it is not extraordinary that cases of homicidal monomania should occasionally appear. So strange are the caprices of the mind, that by giving notoriety or intense interest to any crime, however atrocious, you will produce imitations. Such exhibitions should be suppressed by law, as of a highly dangerous and immoral tendency.

We are desirous of calling attention to the cases of homicidal mania from impulse, to which we have just specially referred, in order to expose the fallacy of a common dictum among our law authorities. It has been repeatedly held by judges that insanity must never be inferred from the act committed; and that the nature and atrocity of the act could not be adduced in support of that plea. We would simply ask, after the occurrence of such cases as those above referred to, how is it possible that a principle of this kind can be maintained as consistent with either law or reason? It cannot be enforced; or if it be, then persons must be executed whose execution could have no effect as an example. In cases of this kind, the act itself is in general the only evidence of insanity: it is said, the admission of such a principle would be dangerous; in our view there is equal, if not greater, danger from its exclusion. In the present state of the law there is always a risk of such persons being executed like sane criminals. (See Greensmith's case, No. XIX. p. 144.)

On the other hand, the plea of insanity may be improperly made and abetted by medical testimony. The act committed may be wrongly taken as evidence of insanity; for it is impossible to say, à priori, what criminal acts should, and what should not, be deemed acts of insanity. This is undoubtedly true; and the only remedy, in our opinion, is to abolish medical testimony as it is at present given on trials for crime where insanity is the plea. Questions of this important nature should be referred to a board of twelve or more competent men: the state of mind of a person accused of crime should not be left to be decided by those members of the profession whom the prisoner or his friends may select for their known support of his case. As to the question of responsibility and punishment, this should be intrusted to the authorities of the law.

---

